# Emergent Protective Organogenesis in Date Palms: A Morpho-Devo-Dynamic Adaptive Strategy during Early Development

**DOI:** 10.1105/tpc.19.00008

**Published:** 2019-05-29

**Authors:** Ting Ting Xiao, Alejandro Aragón Raygoza, Juan Caballero Pérez, Gwendolyn Kirschner, Yanming Deng, Brian Atkinson, Craig Sturrock, Vinicius Lube, Jian You Wang, Gilles Lubineau, Salim Al-Babili, Alfredo Cruz Ramírez, Malcolm Bennett, Ikram Blilou

**Affiliations:** a King Abdullah University of Science and Technology (KAUST), Biological and Environmental Sciences and Engineering (BESE), Thuwal, 23955-6900, Saudi Arabia; b Molecular and Developmental Complexity Group, Unidad de Genómica Avanzada-Laboratorio Nacional de Genómica para la Biodiversidad (LANGEBIO), CINVESTAV, Irapuato, Guanajuato, 36821, México; c Provincial Key Laboratory for Horticultural Crop Genetic Improvement, Institute of Leisure Agriculture, Jiangsu Academy of Agricultural Sciences, Nanjing, 210014, Jiangsu, China; d Hounsfield Facility, School of Biosciences, University of Nottingham, Nottingham LE12 3RD, United Kingdom; e King Abdullah University of Science and Technology (KAUST), Physical Science and Engineering Division, COHMAS Laboratory, Thuwal 23955-6900, Saudi Arabia

## Abstract

Desert plants have developed mechanisms for adapting to hostile desert conditions, yet these mechanisms remain poorly understood. Here, we describe two unique modes used by desert date palms (*Phoenix dactylifera*) to protect their meristematic tissues during early organogenesis. We used x-ray micro-computed tomography combined with high-resolution tissue imaging to reveal that, after germination, development of the embryo pauses while it remains inside a dividing and growing cotyledonary petiole. Transcriptomic and hormone analyses show that this developmental arrest is associated with the low expression of development-related genes and accumulation of hormones that promote dormancy and confer resistance to stress. Furthermore, organ-specific cell-type mapping demonstrates that organogenesis occurs inside the cotyledonary petiole, with identifiable root and shoot meristems and their respective stem cells. The plant body emerges from the surrounding tissues with developed leaves and a complex root system that maximizes efficient nutrient and water uptake. We further show that, similar to its role in Arabidopsis (*Arabidopsis thaliana*), the SHORT-ROOT homolog from date palms functions in maintaining stem cell activity and promoting formative divisions in the root ground tissue. Our findings provide insight into developmental programs that confer adaptive advantages in desert plants that thrive in hostile habitats.

## Abstract (precis)

Date palm uses unique developmental modes to protect its embryo and organs by pausing development during germination and confining developing organs within a multilayered structure in early development

## INTRODUCTION

The process through which a complex adult form emerges from a cascade of developmental events, tightly controlled in space and time, is called morphogenesis. Changes in environmental conditions can modulate this process, thereby providing an adaptive developmental plasticity to overcome environmental challenges. In animals, diapause causes a temporary arrest in development to allow the embryo to survive harsh conditions and to ensure that postnatal development can be completed when environmental conditions become more favorable (Apfeld and Kenyon, 1998; Fielenbach and Antebi, 2008; Fenelon et al., 2014; Liu et al., 2016). In plants, this process is called dormancy during which a fully developed embryo rests inside the seed. Under optimal conditions, the seed germinates and produces a seedling that grows and continuously generates organs that form the adult plant body. Dormancy and diapause are primarily linked to survival in low temperatures during winter. However, high-temperature–induced diapause or dormancy has been observed in worms, insects, and plants (Wadsworth et al., 2013).

**Figure:**
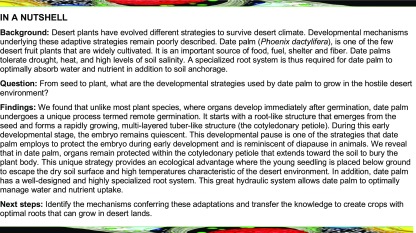


The date palm (*Phoenix dactylifera*) is one of the few fruit trees that, remarkably, can grow in the desert, a habitat with an arid climate where extreme temperature changes and drought conditions limit plant growth. Date fruits are essential for sustaining current desert agriculture production. However, breeding strategies aimed at improving date palm production are hindered by the long generation time (fruit production starts 5 to 10 years after planting) and their dioecious nature (male and female flowers on different trees), in addition to the large phenotypic variation in progeny caused by heterozygosity (El Hadrami et al., 2011).

To cope with changes in their surrounding environment, palms have adopted three morphologically distinct modes of germination (Supplemental Figure 1A; Pinheiro, 2001; Baskin and Baskin, 2014): (1) adjacent ligular: the cotyledon sheath with the embryo is located directly next to the seed; (2) remote ligular: the cotyledon extends to form a petiole connecting the haustorium to the cotyledon ligule; and (3) remote tubular: a petiole forms and becomes a tubular structure instead of a ligule (Demason, 1984, 1988; Henderson, 2006; Iossi et al., 2006; Tillich, 2007). Date palms germinate by the remote tubular mode; germination begins in this case with the emergence of a root-like structure termed the cotyledonary petiole. This structure elongates to form the primary root and a plumule that together with the primary leaf, emerge through an opening/cleavage from the cotyledonary petiole (Demason, 1984, 1988; Iossi et al., 2006; Tillich, 2007).

Later in development, the date palm develops an intricate root system that comprises the main root and the anchor roots that grow vertically, secondary roots that grow laterally, and negatively geotropic roots that grow aboveground (called pneumatophores; Jost, 1887; de Granville, 1974; Seubert, 1997). A root often supports multiple pneumatophores, forming the pneumatorhiza (Seubert, 1997; Dreyer et al., 2010). In date palms, the primary root consists of multiple tissue types: the outermost layers form the rhizodermis/velamen (R/V) that surrounds the exodermis, which in turn surrounds two types of cortex tissues, and the outer cortex and the inner cortex. Inside the inner cortex, where intercellular spaces form the aerenchyma, lies the endodermis that surrounds the vascular cylinder (Drabble, 1904; de Granville, 1974; Seubert, 1997).

A detailed characterization of early developmental programs in date palms and study of their adaptation to arid conditions are currently lacking, despite the body of literature on date palm anatomy and growth (Drabble, 1904; Seubert, 1996, 1997; Pinheiro, 2001; Henderson, 2006; Iossi et al., 2006; Tomlinson, 2006) and the extensive efforts made on date palm genomics, proteomics, and metabolomics (Al-Dous et al., 2011; Al-Mssallem et al., 2013; Marondedze et al., 2014; Hazzouri et al., 2015; Stephan et al., 2018). Addressing these knowledge gaps could provide important foundational knowledge for the expansion of desert agriculture production, which is essential in the face of global climate change.

Here, we present a comprehensive study of the early development of date palms, from germination to seedling stage. Germination in date palms begins with the emergence of the cotyledonary petiole. We found that the undeveloped embryo resides within the growing tip of the cotyledonary petiole. This developmental embryonic pause coincides with a reduced rate of cell division, reduced expression of key developmental genes, and an accumulation of hormones associated with dormancy as well as with responses to abiotic and biotic stresses. Remarkably, we observed that organogenesis occurs within the cotyledonary petiole. The developing seedling remains encapsulated and produces root, shoot, and leaf primordia that express organ-specific genes. As growth continues, the leaf protrudes through the surrounding tissue. The shoot meristem produces new primordia that proliferate and elongate and, in turn, allow the date palm aboveground organs to increase in diameter and height.

We found that as the plant grows, the cotyledonary petiole tip/radicle elongates, giving rise to the future root. This is in contrast to grasses where the root, before penetrating the soil, splits from the coleorhiza, a nonvascularized multicellular embryonic tissue that protects the root (Sargent and Osborne, 1980; Barrero et al., 2009). Our anatomical description of the pneumatophores indicates that these specialized roots have different zones where the cell layers change in number along with the proximal-distal axes. We also reveal that the function of the developmental regulator SHORT-ROOT (SHR) is conserved.

Our findings indicate that date palms have developed unique adaptive developmental strategies to survive in the hostile desert environment by protecting their meristems and organs during early development and by orchestrating an efficient growth where the root system is structurally adapted to maximize water uptake and prevent its loss.

## RESULTS

### Remote Germination

**Figure 1.**
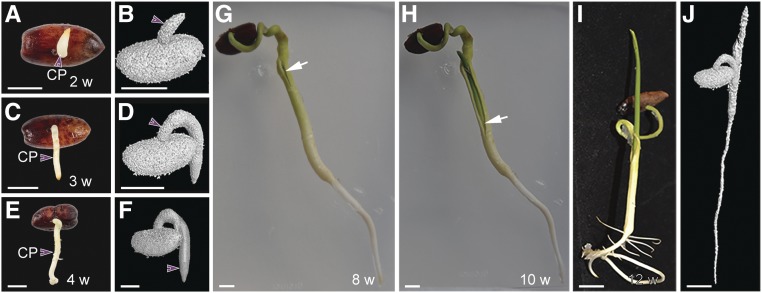
Germination Process in Date Palms. **(A)** to **(J)** Two- to 4 weeks after germination (see **[A]** to **[F]**), 8 weeks after germination **(G)**, 10 weeks after germination **(H)**, and 12 weeks after germination (see **[I]** and **[J]**). **(A)**, **(C)**, **(E)**, and **(G)** to **(I)** are macrophotographs. **(B)**, **(D)**, **(F)**, and **(J)** are images obtained from 3D x-ray computed tomography. The white arrows in **(G)** and **(H)** point to tissue apertures allowing leaves to emerge; purple arrowheads point to the cotyledonary petiole (CP). Images are representative of 40 seedlings. Bar in **(A)** to **(J)** = 1 cm. w, weeks after germination.

In most seed plants species, germination produces a seedling with a discernible shoot and root (Supplemental Figure 1B). In the date palm, this developmental process is described as remote germination during which the seedling develops at a distance from the seed (Supplemental Figure 1A; Demason, 1984; Pinheiro, 2001; Henderson, 2006; Iossi et al., 2006; Baskin and Baskin, 2014). To characterize this remote germination process, we first monitored date palm growth from germination to the seedling stage (Figure 1). Our macroscopic analysis combined with noninvasive x-ray micro-computed tomography (XμCT) revealed that, in date palms, germination occurs in two phases. First, the cotyledonary petiole emerges and develops away from the seed coat during the first few weeks after germination (Figures 1A to 1F; Supplemental Movie). Second, the first leave or plumule appears through a protrusion from the cotyledonary petiole (Figures 1G to 1J). These results suggest that in date palm, organogenesis occurs within the cotyledonary petiole.

**Figure 2.**
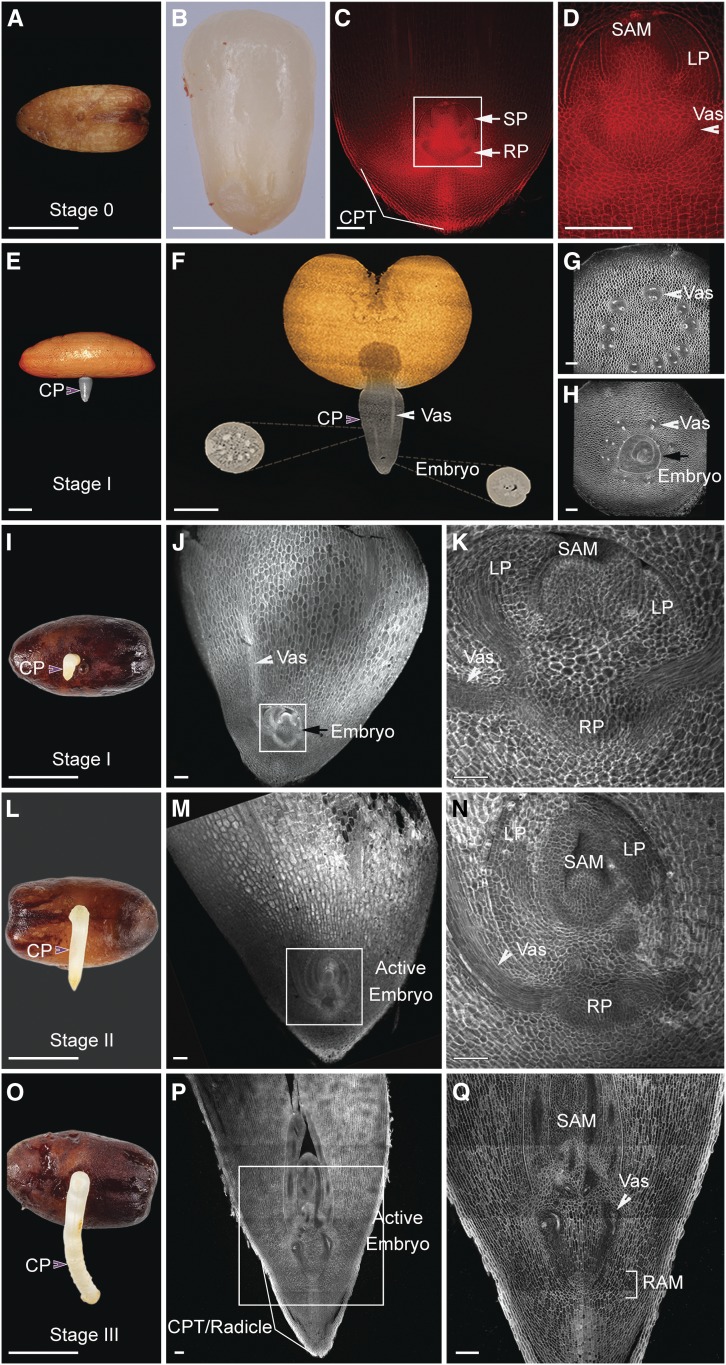
Growth Dynamics during Germination in Date Palms. **(A)** Date palm seed containing the embryo. Bar = 1 cm. **(B)** Embryonic sac dissected from the seed (*n* = 20). Bar = 0.5 cm. **(C)** and **(D)** Longitudinal section of dissected embryo sac (see **[C]** and **[D]**) stained with mPS-PI (*n* = 20). The white arrows indicate root and shoot axes in **(C)**. Bar in **(C)** and **(D)** = 100 µm. **(E)** and **(F)** 3D image of germinated date palm at stage I imaged with XμCT (*n* = 3). Bar in **(E)** = 5 mm; bar in **(F)** = 2 mm. **(G)** and **(H)** Confocal images of cotyledonary petiole sections taken at the same stage as in **(F)**. The section in **(G)** at the top part, which does not include the embryo; the section in **(H)** includes a transverse view of the embryo (*n* = 15). Bar in **(G)** and **(H)** = 100 µm. **(I)** to **(Q)** Date palm growth at 1 to 4 weeks after germination (*n* = 25). **(I)**, **(L)**, and **(O)** are macrophotographs; **(J)**, **(K)**, **(M)**, **(N)**, **(P)**, and **(Q)** are confocal images of longitudinal vibratome sections with the cell walls stained with SCRI Renaissance 2200 (*n* = 10). White arrowheads point to the vasculature (Vas); purple arrowheads point to the cotyledonary petiole (CP). The black arrows in **(H)** and **(J)** indicate the embryo. Images are representative of the total number (*n*) of seedlings that were studied. **(D)**, **(K)**, **(N)**, and **(Q)** are insets of **(C)**, **(J)**, **(M)**, and **(P)**, respectively. Bar in **(I)**, **(L)**, and **(O)** = 1 cm; bar in **(M)** and **(P)** = 100 µm; bar in **(K)** and **(N)** = 50 µm CPT, cotyledonary petiole tip; LP, leaf primordia; RAM, root apical meristem; RP, root pole; SP, shoot pole.

**Figure 3.**
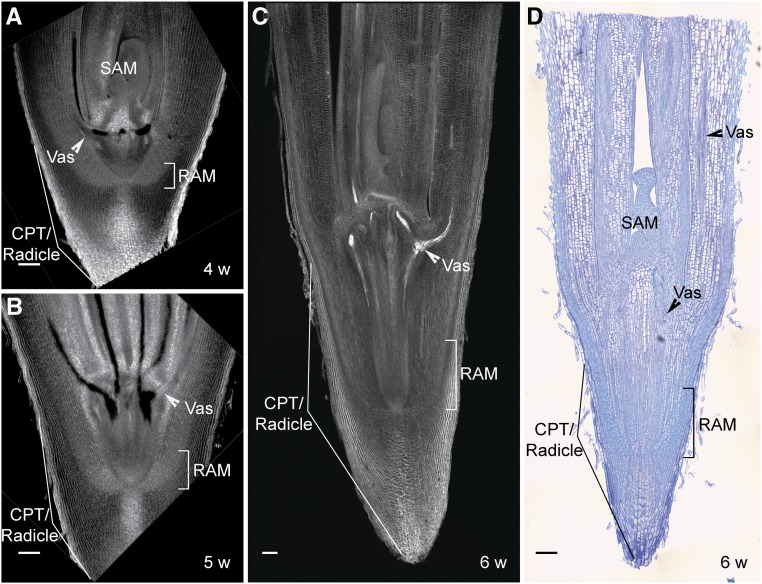
Encapsulated Organogenesis in Date Palms. **(A)** to **(C)** Confocal images of 4- to 6-week-old longitudinal sections of date palm stained with mPS-PI. **(D)** Differential interference contrast images of the longitudinal plastic section stained with toluidine blue O. Images are representative of 10 seedlings that were studied. Bar = 100 µm for **(A)** to **(D)**. CPT, cotyledonary petiole tip; ESJ, root–shoot junction; RAM, root apical meristem; SAM, shoot apical meristem; Vas, vasculature; w, weeks after germination.

To validate these observations, we analyzed the cellular structures of the embryo isolated from the seed (stage 0) and within the cotyledonary petiole at different stages after emergence (stages I to III). Interestingly, we found no anatomical differences between stage 0 and stage I, confirming that during these stages, embryo development was paused (Figures 2A to 2K). At stages II and III, embryo growth is resumed, resulting in a small seedling encapsulated within the cotyledonary petiole and where the root and shoot meristems and developing leaf primordia are morphologically and spatially distinguished (Figures 2L to 2Q). Later in development, the cotyledonary petiole tip/radicle elongates, forming the main root (Figures 3A to 3D).

**Figure 4.**
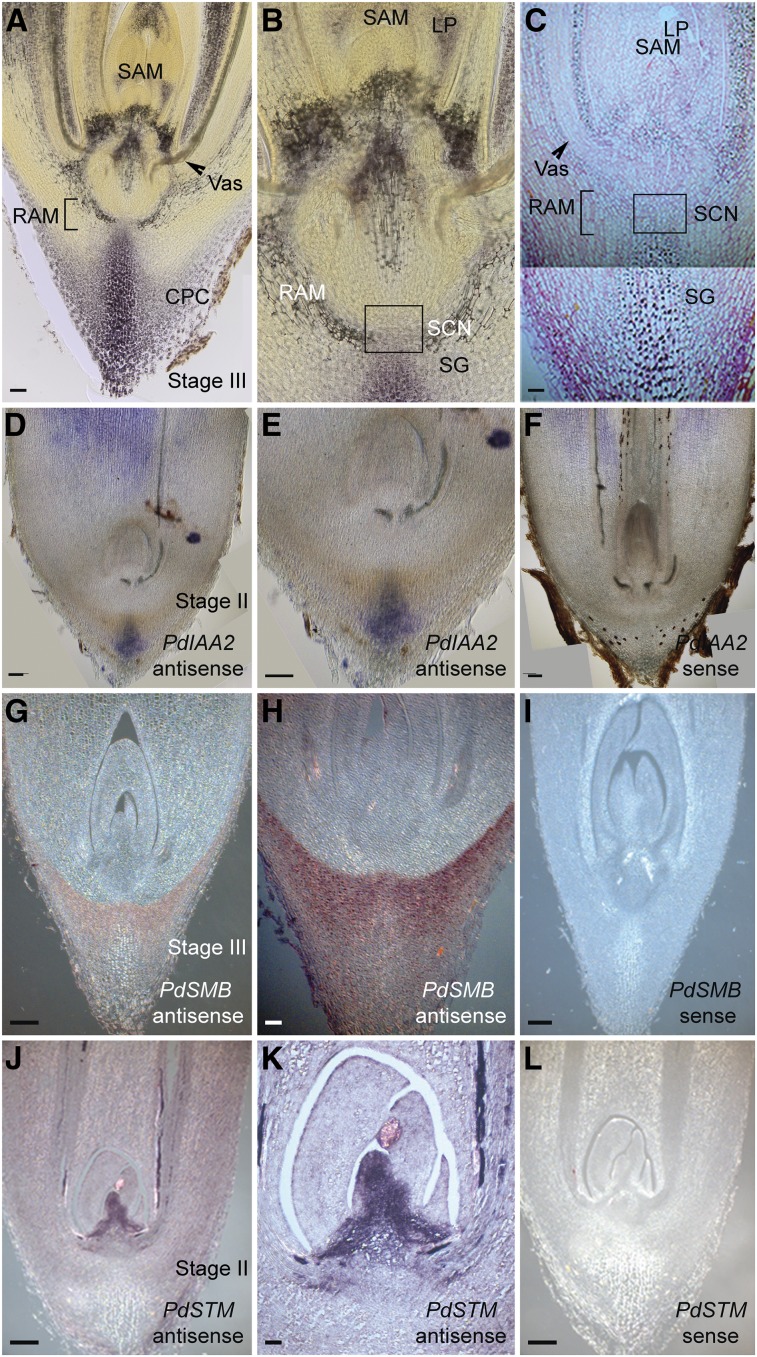
Characterization of Root and Shoot Meristem in Date Palms. **(A)** to **(C)** Accumulation of starch granules at the root tip. Light microscope images of vibratome sections stained with Lugol (see **[A]** and **[B]**; *n* = 8; brown color). Plastic longitudinal sections stained with ruthenium red (pink color) and Lugol (see **[C]**; *n* = 8). Bar in **(A)** and **(C)** = 50 µm. CPC, cotyledonary petiole cap; LP, leaf primordia; RAM, root apical meristem; SCN, stem cell niche; SG, starch granules. **(D)** to **(F)** Gene expression analysis with in situ hybridization using the date palm auxin response gene *IAA2* (*PdIAA2*); antisense probe (see **[D]** and **[E]**; *n* = 15; mRNA signal is shown in blue/purple); sense control (see **[F]**; *n* = 15). Bar in **(D)** to **(F)** = 100 µm. **(G)** to **(I)** Differentiated columella layers are marked with *PdSMB*. The antisense probe (see **[G]** and **[H]**; *n* = 10; mRNA signal is shown in brown) is compared with the sense control for *PdSMB* (see **[I]**; *n* = 10). Bar in **(G)** and **(I)** = 200 µm. **(J)** to **(L)** The shoot meristem is marked with *SHOOT MERISTEMLESS* gene *STM* (*PdSTM*), the antisense probe (see **[J]** and **[K]**; *n* = 9; mRNA signal is shown in brown); sense control for *PdSTM* (see **[L]**; *n* = 9). **(E)**, **(H)**, and **(K)** are zoomed in from **(D)**, **(G)**, and **(J)**, respectively. Images are representative of the total number (*n*) of seedlings that were studied. Bar in **(J)** and **(L)** = 200 µm; bar in **(H)** = 100 µm; and bar in **(K)** = 50 µm.

Next, we sought to determine whether the encapsulated seedling (stages II and III) displayed structures with root and shoot characteristics. In plant roots, the columella layers accumulate starch granules, allowing the positioning of the stem cell niche (Dolan et al., 1993; Scheres et al., 1994; Kirschner et al., 2017). Starch granules, together with auxin accumulation at the distal tip, play a key role in sensing and directing a root’s responses to gravity (Sabatini et al., 1999; Ottenschläger et al., 2003). In date palms, we found an accumulation of starch granules at the distal root tip of the cotyledonary petiole (Figures 4A to 4C). This accumulation correlated with high mRNA levels of the auxin response gene *INDOLE-3-ACETIC ACID INDUCIBLE2* (*PdIAA2*; Swarup et al., 2007) in the differentiated columella cells, consistent with the auxin maxima present at the distal tip (Figures 4D to 4F).

The NAC domain gene *SOMBRERO* (*SMB*) also marks the mature columella cells in Arabidopsis (*Arabidopsis thaliana*) and has been described to control the division rates and the orientation of the cell division plane of the columella and epidermis/lateral root cap stem cells (Willemsen et al., 2008). Similarly, we detected *PdSMB* in the differentiated columella layers and the root cap region of the cotyledonary petiole (Figures 4G to 4I). These data indicate that the distal tip of the cotyledonary petiole exhibited root tip characteristics.

We also assessed whether the shoot identity genes were expressed at the apical pole of the encapsulated seedling. mRNA of the date palm ortholog for *SHOOT MERISTEMLESS* (*PdSTM*; Long et al., 1996) was confined to the apical region that coincides anatomically with the shoot apical meristem (SAM; Figures 4J to 4L), whereas the *CUP-SHAPED COTYLEDON1* ortholog (Aida et al., 1997) mRNA was detected at the boundary between the SAM and the emerging leaf primordia (Supplemental Figures 2A to 2C). The *AINTEGUMENTA* gene (Mudunkothge and Krizek, 2012) was expressed in the SAM and developing leaves (Supplemental Figures 2D to 2F).

### Protective Organogenesis in Date Palm

**Figure 5.**
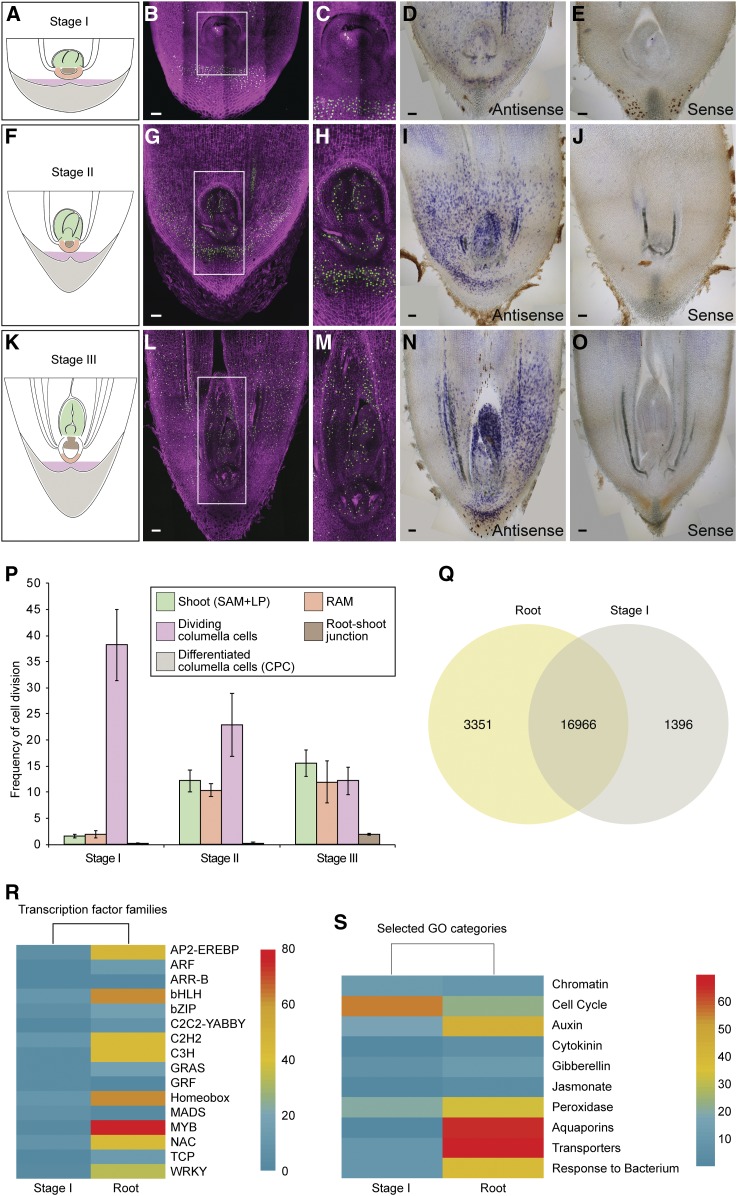
Postembryonic Development Correlates with an Increase in Cell Division Rates and Activation of Developmental Genes. **(A)** to **(O)** Cartoons (see **[A]**, **[F]**, and **[K]**) representing the early developmental stages of date palms: stage I (see **[A]** to **[E]**), stage II (see **[F]** to **[J]**), and stage III (see **[K]** to **[O]**). Confocal images (see **[B]**, **[C]**, **[G]**, **[H]**, **[L]**, and **[M]**) of vibratome sections from the cotyledonary petiole showing dividing cells as captured by EdU staining (*n* = 35). Dividing EdU-stained nuclei are shown in green; nuclei counterstained with Hoechst 33258 are shown in magenta. **(C)**, **(H)**, and **(M)** are inset from **(B)**, **(G)**, and **(L)**, respectively. All bars = 100 µm. In situ hybridization (see **[D]**, **[E]**, **[I]**, **[J]**, **[N]**, and **[O]**) showing *PdHistone-H4* expression (*n* = 20; signal is shown in blue/purple color). **(B)** to **(E)**, **(G)** to **(J)**, and **(L)** to **(O)** are representative images of the total number (*n*) of seedlings that were studied. **(P)** Graph showing cell division rates in different zones and at different developmental stages of the seedlings. The *x* axis represents developmental stages, and the *y* axis represents the measured frequency of cells incorporating EdU. Each column represents the average cell division frequency in a specific zone of the tip of the cotyledonary petiole. Error bars represent ±se. Numbers (*n*) of seedlings that were used: stage I, *n* = 14; stage II, *n* = 12; and stage III, *n* = 6. Average nuclei counted (Hoechst) for the cell division frequency calculation per stage and per zone are represented as shoot:RAM:dividing columella:root–shoot junction as follows: 604:210:342:97 (stage I), 557:275:376:105 (stage II), and 597:494:271:107 (stage III). Zones are indicated in the following colors in **(A)**, **(F)**, and **(K)**: Shoot (green); RAM (orange); dividing columella (pink); root–shoot junction (brown); cotyledonary petiole cap (grey). Measured zones are represented in **(P)**. **(Q)** Venn diagram showing overlapping and differentially expressed genes in stage I and 12-week-old roots resulting from RNA-seq data. **(R)** Heatmap representation of differentially expressed developmental genes. **(S)** Selected categories of enriched GO. Upregulation is shown in red, downregulation is shown in blue. CPC, cotyledonary petiole cap; CPT, cotyledonary petiole tip; LP, leaf primordia; RAM, root apical meristem.

**Figure 6.**
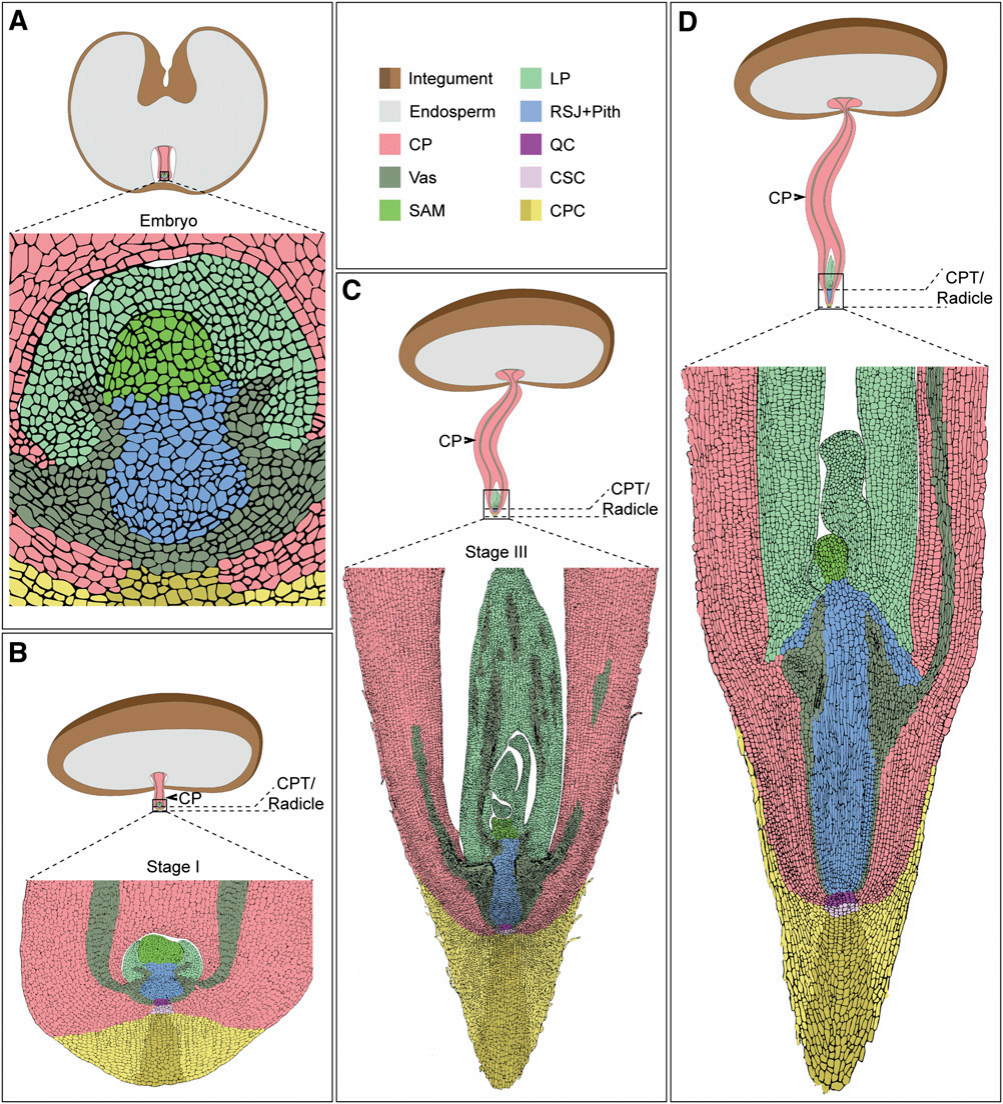
Schematic Representations of Date Palm Development from Embryo to Forming Organs within the Cotyledonary Petiole. Representations are illustrations from longitudinal sections of real samples. Black arrowhead points to the cotyledonary petiole (CP). CPC, cotyledonary petiole cap; CSC; columella stem cells; LP; leaf primordia; QC; quiescent center; RSJ+pith, root–shoot junction including the pith; Vas; vasculature.

Our initial observations revealed that the growing cotyledonary petiole contained an embryo with paused growth that resumed development a few weeks after emerging from the seed. To evaluate these developmental dynamics, we monitored the division rates from stage I to stage III using ethynyl deoxyuridine (EdU), which incorporates into newly synthesized DNA and can be monitored by a fluorescent dye (Cruz-Ramírez et al., 2013). At stage I, fluorescence was mainly observed in the tissue layers surrounding the embryo (Figures 5A to 5C), whereas only a few cells in the embryo displayed fluorescence, indicating a low rate of cell division in the embryo (Figure 5P). At stages II and III, more cells in the embryo showed fluorescence, which correlated with an increase in the frequency of cell division throughout the embryo (Figures 5F to 5H, and 5P) and the newly developed leaf primordia (Figures 5K to 5M, and 5P). The surrounding layers continue to divide and grow in response to gravity, carrying the embryo away from the seed (Figure 6).

To relate the observed cell division rates to cell cycle activity, we tested the accumulation of mRNA of the cell cycle gene *HISTONE-H4*, which marks the G1/S phase during the cell cycle (Gutierrez, 2009; Kirschner et al., 2017). We found that *PdHISTONE-H4* mRNA accumulated in a few cells of the embryo at stage I (Figures 5D and 5E). *PdHISTONE-H4* expression expanded and became evenly distributed throughout the subsequent developmental stages (Figures 5D, 5E, 5I, 5J, 5N, and 5O). Our data show that in stage I growth of the embryo paused (Figures 6A and 6B). From stage II onward, organogenesis took place within the cotyledonary petiole where the growing embryo/young seedling remained attached to the maternal tissue (Figures 6C and 6D).

Since embryonic dormancy is modulated by growth and stress hormones (Barrero et al., 2009; Schiesari and O’Connor, 2013), we measured hormone levels in the cotyledonary petiole in stage I and in the seedling, once it had emerged from the cotyledonary petiole, where the root, shoot, and surrounding sheet could be easily distinguished and dissected separately (Supplemental Figure 3). In stage I, we observed high levels of abscisic acid (ABA; Supplemental Figure 3), which correlates with its role in repressing cell division and inducing embryonic arrest (Sondheimer et al., 1968; Barlow and Pilet, 1984). In the developed seedlings, we found a significant decrease in ABA in the shoot and cotyledonary petiole, in addition to an increase in gibberellic acid (GA) levels in the roots (Supplemental Figure 3). In both stage I and the seedlings, we detected an accumulation of the defense hormones salicylic acid (SA) and jasmonic acid (JA; Supplemental Figure 3).

### Comparative Transcriptome Analysis of Embryos and Roots Shows Distinct Gene Clusters

To identify transcriptional determinants that govern developmental decisions and that confer adaptation to desert conditions, we conducted a comparison of the transcriptomes of date palm embryos and root tissues. RNA sequencing (RNA-seq) reads were generated from total RNA isolated from the emerging cotyledonary petiole in stage I and from root tissues of emerged seedlings. Our cross-comparison of differentially expressed genes between stage I and the root tips (using a fold-change of >2× and a false discovery rate [FDR] of <0.05) revealed 1396 transcripts that were highly and specifically expressed in stage I, 3351 transcripts that were highly and specifically expressed in root tips, and an overlap of 16,966 transcripts observed in both organs (Figure 5Q).

Next, we performed gene ontology (GO) analysis to annotate the function of differentially expressed genes in embryos and roots, and we categorized them by their function either in development, biotic/abiotic resistance, or metabolism. We found an enrichment in expression of developmental genes including transcription factor families in the roots compared with those in the embryo (Figure 5R), which is consistent with the paused development in stage I. Among the highly enriched genes in roots were those coding for aquaporins (Figure 5S), proteins involved in facilitating water transport and ion movement in response to osmotic stresses (Gambetta et al., 2017). The enrichment of genes involved in responding to bacteria in roots (Figure 5S) reflects the importance of roots associating with bacterial communities, which might have a role in promoting date palm resistance to drought and salinity (Cherif et al., 2015).

### Organization of the Date Palm Root Meristem

**Figure 7.**
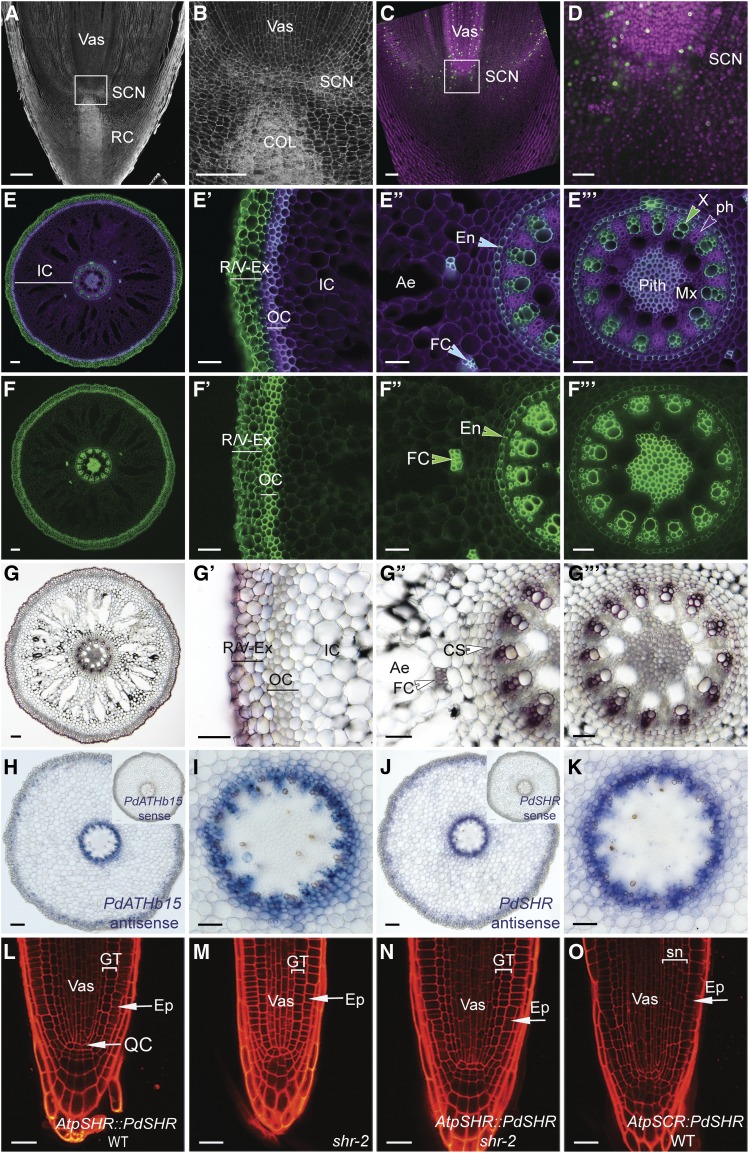
Date Palm Root Anatomy. **(A)** and **(B)** Confocal scanning images of longitudinal sections of roots obtained from 7-month-old plants and stained with mPS-PI (*n* = 3). **(C)** and **(D)** Dividing nuclei are stained with EdU (green; *n* = 5). Nuclei were counterstained with Hoechst (purple). **(E)** to **(F′′′)** Confocal image of a cross section of a root stained with SCRI Renaissance 2200. Purple indicates cell wall staining; green is the auto-fluorescence marking lignin and suberin deposition within the roots (see **[E]** to **[E′′′]**). A berberine-stained cross section marks suberin in green (see **[F]** to **[F**′′′]**)**. **(G)** to **(G′′′)** Lignin accumulation in a cross-section stained with phluoglucocinol in dark brown. **(H)** to **(K)** RNA in situ hybridization in 12-week-old roots. Signal blue/purple shows mRNA localization of vascular *PdATHB15* (see **[H]** and **[I]**) and *PdSHR* (see **[J]** and **[K]**) in date palm roots. Date palm SHR function is conserved in Arabidopsis. **(L)** to **(O)** Confocal images of a root stained with PI of At*pSHR*:*PdSHR* in the wild type (WT; see **[L]**; *n* = 6); *shr* mutant (see **[M]**; *n* = 6), At*pSHR*:*PdSHR* in *shr* (see **[N]**; *n* = 8), and *AtpSCR*:*PdSHR* in wild type (see **[O]**; *n* = 6). **(B)**, **(D)**, **(E**′)**** to **(E**′′′)****, **(F**′)**** to **(F**′′′)****, **(G**′)**** to **(G**′′′)****, **(I)**, and **(K)** show zoomed images from **(A)**, **(C)**, **(E)**, **(F)**, **(G)**, **(H)**, and **(J)**, respectively. Bar in **(A)**, **(C)**, **(E)**, **(F)**, **(G)**, **(H)**, and **(J)** = 100 μm; bar in **(B)**, **(D)**, **(E**′)**** to **(E**′′′)****, **(F**′)**** to **(F**′′′)****, **(G**′)**** to **(G**′′′)****, **(I)**, and **(K)** = 50 μm; and bar in **(L)** to **(O)** = 20 µm. Images are representative of the total number (*n*) of roots that were studied. Ae, aerenchyma; Col, columella; CS, Casparian strips; En, endodermis; Ep, epidermis; Ex, exodermis; FC, fiber cells; GT, ground tissue: cortex and endodermis, IC, inner cortex; Mx, metaxylem; OC, outer cortex; Ph; phloem; SCN, stem cell niche; Sn, supernumerary layers; Vas, vasculature; X, xylem.

As the date palm root is adapted to the desert environment, we next explored whether this adaptation is evident in the cellular organization of the date palm roots. We first analyzed the root tip of a 7-month-old plant and used both Lugol and modified pseudo-Schiff propidium iodide (mPS-PI; Truernit et al., 2008) stains to visualize starch in the differentiated columella root cells in longitudinal root sections (Figures 7A and 7B; Supplemental Figures 4A and 4B). We found that, above the stained columella layers, three to four cell layers did not contain starch granules and these layers are likely to form the root stem cell niche (Figures 7A and 7B; Supplemental Figures 4A and 4B). EdU staining showed limited cell division rates around the quiescent center region and the differentiated columella cells (Figures 7C and 7D).

**Figure 8.**
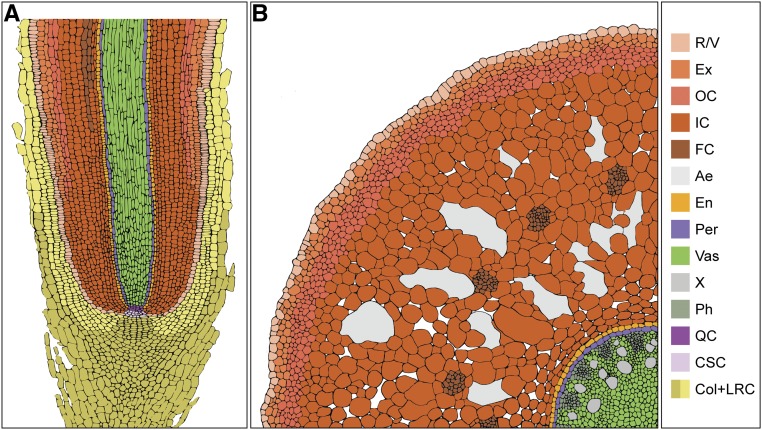
Schematic Representation of the Date Palm Root. **(A)** and **(B)** Longitudinal section **(A)** and cross section **(B)**. Colors represent distinct tissue types. Ae, aerenchyma; Col+LRC, columella and lateral root cap; CSC, columella stem cells; En, endodermis; Ex, exodermis; FC, fiber cells; IC, inner cortex; OC, outer cortex; Per, pericycle; Ph; phloem; QC, quiescent center; X, xylem; Vas, vasculature.

Date palm roots have been described as having multilayered lignified tissues (Drabble, 1904; de Granville, 1974; Seubert, 1997). To evaluate precisely the composition of these tissue layers, we performed histochemical analysis in tissue sections (Figures 7E to 7G; Figure 8). We found that suberin accumulated in the outermost layers (R/V, exodermis) and outer cortex (Figures 7E, E′, F, and F′). Lignin accumulation was only observed in the R/V and exodermis (Figures 7G and G′). The inner layers forming the inner cortex contained larger cells with intercellular air spaces termed aerenchyma (Figures 7E to 7E′′, F, F′′, and G to G′′). Within the cortex, we found bundle cells that accumulated suberin and lignin (Figures 7E′′, F′′, and G′′). A single endodermal layer with Casparian characteristics (Figures 7E to 7E′′′, 7F to 7F′′′, and 7G to G′′′) encircled a vascular system composed of suberized and lignified xylem and phloem cells expressing the vascular gene *PdATHB15* (Figures 7H and 7I). Cells at the center forming the pith accumulated more suberin than lignin (Figures 7E′′′, 7F′′′, and 7G′′′). The accumulation of suberin and lignin in different tissue layers of date palm roots provides an adaptive advantage by preventing water loss and altering ion transport pathways.

### A Radial Gene Network Is Functionally Conserved between Date Palm and Arabidopsis

In Arabidopsis, the cell fate determinant SHR regulates endodermal patterning (Helariutta et al., 2000). To assess whether the SHR ortholog in date palm, *PdSHR*, performs a similar function, we first analyzed the localization of mRNA in its roots by in situ hybridization and found that, like in Arabidopsis and rice, *PdSHR* is transcribed in the vasculature (Figures 7J and 7K). Furthermore, we detected *PdSHR* in the endodermis, cortex, and fiber cells of older and mature roots (Figure 7J; Supplemental Figure 4D). To assess whether *PdSHR* fulfilled a similar function in endodermal specification, we introduced the *PdSHR* driven by the Arabidopsis *SHR* promoter (*AtpSHR*) in the wild type and in *shr* mutants lacking the endodermal layer. We found that *AtpSHR*:*PdSHR* complemented the root length and restored the double-layered ground tissue in *shr* mutants (Figures 7L to 7N). In Arabidopsis, when *SHR* is expressed from the Arabidopsis *SCARECREOW* promoter *AtpSCR*, additional layers are produced in the ground tissue (Sena et al., 2004). Similarly, *PdSHR* was also able to produce multiple ground tissue layers in Arabidopsis when expressed from *AtpSCR* (Figure 7O). These data suggest that, despite being phylogenetically distant, the radial patterning network is conserved between date palm and Arabidopsis.

### XμCT Reveals Geotropic Pneumatophores within the Root System

**Figure 9.**
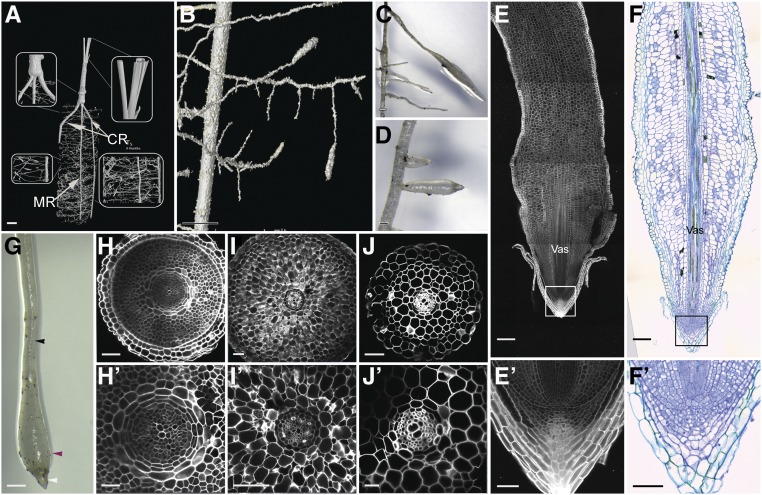
Characterization of Date Palm Pneumatophores. **(A)** and **(B)** XµCT images showing the root system architecture of the date palm: the primary root with secondary lateral horizontal growth and aerial roots with upward vertical growth. **(B)** is a zoomed image of **(A)**. **(C)**, **(D)**, and **(G)** Micrographs showing pneumatophores (*n* = 10). **(E)** and **(E**′)**** Longitudinal sections of date palm pneumatophores stained with mPS-PI (*n* = 5). **(F)** and **(F**′)**** DIC images of sections stained with toluidine blue O (*n* = 5). **(H)** to **(J**′)**** Cross sections of different zones within the pneumatophore (*n* = 5); basal thin region (white arrowhead; see **[H]** and **[H′**]**)**, basal thick region (purple arrowhead; see **[I]** and **[I**′]****), and apical thin region (black arrowhead; see **[J]** and **[J**′]****). **(E**′)****, **(F**′)****, **(H**′)****, **(I**′)****, and **(J**′)**** are enlargements of **(E),** **(F),** **(H),** **(I),** and **(J),** respectively. Images are representative of the total number (*n*) of roots that were studied. Bar in **(A)** = 20 mm; bar in **(B)** = 4.5 mm; bar in **(C)**, **(D)**, and **(G)** = 1 cm; bar in **(E)** and **(F)** = 100 µm; bar in **(H)**, **(I)**, **(J)**, **(E**′)****, and **(F**′)**** = 50 µm; and bar in **(H**′)****, **(I**′)****, and **(J**′)**** = 25 µm. CR, crown root; MR, main root.

To characterize the root system architecture of living date palms noninvasively, we followed its development using XμCT (Figure 9A; Supplemental Movie). Time-course CT imaging revealed strongly positive gravitropic growth of the cotyledonary petiole and the later-emerging crown roots. We also observed, in addition to main roots, crown roots, lateral roots, and agravitropic tubular polyp-like structures (Figures 9B to 9J′). These structures are termed pneumatophores (Jost, 1887; Seubert, 1997).

As the pneumatophores showed different root thickness along the proximal-distal axis, we questioned whether these differences resulted from an increase in cell numbers and tissue layers, or from an increase in cell size. Longitudinal and cross-section analyses revealed that along the proximal distal axis, the pneumatophores have zones with distinct widths: the distal tip containing the stem cell niche and the columella layers (Figures 9E′ and 9F′). Above this region resides a thick zone consisting of multiple tissue layers (up to 14 layers from outside to inside; Figures 9E and 9H) containing small cells possibly forming the meristem, a thicker zone with less tissue layers composed of larger cells (up to 11 layers from outside to inside; Figure 9I), and a thinner zone with less layers and larger cells (up to seven layers from outside to inside; Figure J). We also observed differences in cell size and number within the vascular tissue (Figures 9H′ and 9J′). These data indicate that unlike most plants’ roots, where the tissue layers are constant along the growth axis, in the pneumatophores both the cell number and the cell size vary along the proximal-distal and radial axes (Figures 9E to 9J′).

## DISCUSSION

Our study highlights important strategies used by the date palm to adapt to the desert environment and reports unique adaptive developmental processes. Remote germination (Figure 1; Demason, 1984, 1988; Iossi et al., 2006; Tillich, 2007) encapsulates the growing embryo within the growing cotyledonary petiole that penetrates the soil, deeply burying the meristems and the newly formed organs. This remarkable developmental program protects the future seedlings from the harsh surrounding desert environment. Furthermore, the embryo development pauses during early germination. This phenomenon is reminiscent of some *Drosophila* species, where photoperiod-mediated reproductive diapause/dormancy in late summer induces the arrest of ovarian development in females at a specific stage. Reproductive development then continues when days become longer and warmer in spring (Salminen et al., 2015).

Our XμCT imaging, combined with tissue sections at different stages, revealed that in date palms, seedlings are encapsulated during early development and form organs while remaining connected to the maternal tissue through the vasculature to provide the water and nutrients necessary to sustain the embryo until emergence (Figure 6). Our data suggest that this process is modulated, at least in part, by hormone homeostasis, illustrated by the increased levels of ABA. Elevated levels of ABA are associated with postponed growth in the early stage, while later stages contain more GA, a phytohormone involved in promoting growth during root development (Supplemental Figure 3). Collectively, our data reflect a mechanism of adaptation to the external environment whereby the embryo and the developing seedlings protect their meristems mechanically and molecularly from heat, drought, and pathogens.

The Arabidopsis root has a single endodermal layer, the formation of which is tightly controlled by confining the movement of the transcription factor SHR to the endodermis (Nakajima et al., 2001; Long et al., 2015). Our data show that the date palm ortholog *PdSHR* was sufficient to rescue the Arabidopsis *shr* mutants and was able to produce extra layers when expressed under the *SCARECROW* promoter (Figure 7L to 7O). These observations indicate that the network controlling root radial patterning is conserved not only in rice (*Oryza sativa*) and Arabidopsis but also in distant species such as the date palm. Interestingly, analysis of *PdSHR* expression in date palm roots revealed a broader expression domain as the mRNA accumulated in the endodermis and the vasculature, and a subset of cells in the cortex including the fiber bundles in the date palm roots. This expression suggests that *PdSHR* function might not be restricted to the specification of the endodermis and may include fiber-cell specification. These fibers display similar features to the vascular tissue, including suberin accumulation and SHR expression, which could imply a function in maximizing efficient water transport and aiding in preventing water loss.

Our XμCT imaging during date palm root development revealed secondary roots similar to lateral roots, and a subset of roots, the pneumatophores, with different diameters along their longitudinal axis growing either horizontally or upward against gravity (Figures 9A and 9B; Jost, 1887; de Granville, 1974; Seubert, 1997). Our imaging showed that the number of cell layers is not constant along the longitudinal axis, and the difference in cell size and cell layers between the different regions of the pneumatophore suggests a different mechanism for the longitudinal zonation pattern when compared with classical lateral roots (Figures 9E to 9F′, 9H to 9J′). One plausible explanation is that the decrease of the layers at the proximal zone might be due to the detachment of the outer layers as the root grows away from the meristem. Another scenario might involve distinct hormone distribution between the zonation leading to this difference in cell size and tissue layers. Pneumatophores can also be found in many other species including mangrove (Yampolsky, 1924), where they contribute to root respiration. It remains to be determined whether pneumatophores have a similar role in date palms. Plausibly, these structures are used to increase the spatial distribution of the roots, not only allowing efficient colonization of the soil area but also maximizing water uptake during sporadic and unexpected rainfall in the desert. During such rainfall events, the pneumatophores, growing near the soil surface, would increase root water uptake, while water retention within the main root would be performed by the suberized outer layers and fiber cells. Understanding the molecular and hormonal mechanisms of pneumatophore formation and function will be useful to engineer crops that can efficiently manage water uptake.

The observed structures in root tissues account for the adaptation of date palms to drought and high salinity. Highly suberized and lignified tissue layers provide an advantage in balancing ion fluxes and monitoring the passage of ions through the vasculature. Furthermore, our transcriptomic data show an enrichment of aquaporins (Figure 5S) in root tissue, which is consistent with their role in promoting root water uptake (Gambetta et al., 2017; Tyerman et al., 2017). We also observed an enrichment in genes involved in response to bacteria. This reflects the importance of root-associated bacterial communities, which have been reported to facilitate nutrient uptake and promote resistance to drought and salinity in date palms (Cherif et al., 2015; Mosqueira et al., 2019). It would be attractive to decipher how the date palm microbiome influences the developmental programs and contributes to these adaptive strategies.

Our data reveal a unique developmental plasticity in date palms that allows them to adapt to their arid environment. Revealing these developmental adaptations provides important foundational knowledge not only for the development of desert agriculture but also for potentially generating crops with an optimized and efficient root system and an increased stress tolerance, which will be essential as the world faces rapid global climate changes.

## METHODS

### Date Palm Seed Sterilization and Germination

Date palm (*Phoenix dactylifera*) seeds were sterilized by washing with detergent (20% [v/v] in water) for 15 min followed by overnight stratification using 20% (v/v) sulfuric acid. The seeds were then washed with sterile water and sterilized with 10% chlorine bleach for 20 min followed by extensive washing with sterile water. The sterilized seeds were plated in 1/2-strength Murashige and Skoog medium containing 0.05% (w/t) morpholinoethanesulfonic acid monohydrate, pH 5.7, 1.0% Suc, and 0.8% plant agar and germinated at 32°C in the dark.

### Tissue Sections, Staining, and Microscopy

Fresh samples were embedded in agarose and sectioned either by hand or by using a VT1000S vibratome (Leica Microsystems). SCRI Renaissance 2200 (SR2200; Musielak et al., 2015) stain was used to visualize cell walls, while berberine hemisulfate stain was used to visualize suberin and lignin (Musielak et al., 2015. Sections were imaged using an LSM 710 upright confocal microscope with excitation of 405 nm for SCRI stain or 488 nm for berberine.

Lignin staining was performed using phloroglucinol-Cl as described by Hofhuis et al. (2016); images were taken using an Olympus BX41 upright bright-field microscope. For longitudinal sections, fresh samples were embedded in 13% low melting agar and sectioned. Lugol was used to visualize starch granules. Samples were mounted in Visikol optical clearing agent and analyzed by an Olympus BX41 upright bright-field microscope.

mPS-PI staining was performed as described previously (Truernit et al., 2008). PI was excited with a 561 nm argon laser with emission detection at 566 to 718 nm.

#### Microtome Plastic Section

Samples were fixed under vacuum for 48 h with 4% paraformaldehyde (w/v) and 5% glutaraldehyde (v/v) in 50 mM phosphate buffer, pH 7.2. Tissue processing and embedding for plastic and paraffin sectioning were performed as described by Long et al. (2015). Root sections were made using an RJ2035 microtome (Leica Microsystems), stained in ruthenium red for 10 min, and then mounted in Depex. Images were captured with a Normaski microscope (Axio Imager; DM5500B microscope, Carl Zeiss).

### EdU Staining for Cell Proliferation Analysis

Cell division rates in date palm seedlings were evaluated using the Click-iT EdU Alexa Fluor 488 imaging kit (C10637, Invitrogen), as described by Cruz-Ramírez et al. (2013). Samples were incubated with EdU diluted in Murashige and Skoog medium for 24 h, fixed in 3.7% formaldehyde for 1 h under vacuum, and then sectioned by vibratome. Sections were permeabilized with PBS containing 0.5% Triton X-100 for 1 h and incubated for 1 h in the dark with a click-it-reaction cocktail that was prepared according to the manual, followed by DNA counterstaining using Hoechst 33342 in PBS under vacuum in the dark for 1 h. Sections were mounted in clearing solution and incubated in the dark for 2 weeks at 4°C as described by Kirschner et al. (2017). Images were captured by LSM 710 inverted confocal microscope (Carl Zeiss).

### Image Acquisition

To obtain high-resolution images from large date palm samples, several images were taken from the same sample and a full image was reconstituted. In the confocal microscope, images were acquired using the tile scan function in the Zen software with automatized stitching. Regions of interest were divided into multiple tiles and imaged individually. The tiles were then combined via automatic stitching to create a large overview image. Images acquired using light microscope were stitched using the Photomerge function in Adobe Photoshop CC 2018.

### RNA in Situ Gene Expression Assays

In situ hybridization was performed using microtome for either tissue sections as described by Carlsbecker et al. (2010), or a whole-mount protocol using vibratome sections. Date palm sequences were retrieved either from the National Center for Biotechnology Information (NCBI) database based on homology with Arabidopsis (*Arabidopsis thaliana*) or from the obtained RNA-seq data set. Probes were amplified from cDNA synthesized from date palm seedlings using the primers listed in Supplemental Table 1 and cloned into the pGEM-T vector (Promega). Probe synthesis was performed as described by Blilou et al. (2002). Samples were imaged using a DM2500 light-emitting diode stand (Leica Microsystems).

### Hormone Measurements from Date Palm Tissues

Phytohormones were quantified according to Delatorre et al. (2017), with the following modifications. Approximately 10 mg of freeze-dried ground tissues was used for the measurements. The internal standards D6-ABA (0.42 ng), D2-GA1 (0.04 ng), D4-SA (0.05 ng), and D2-JA (0.74 ng) were spiked into the ground tissues along with 1.5 mL of methanol. The mixture was sonicated for 15 min in an ultrasonic bath (model 3510, Branson), followed by centrifugation for 10 min at 14,000 rpm at 4°C. The supernatant was collected, and the pellet was re-extracted with 1.5 mL of the same solvent. Next, the two supernatants were combined and dried under vacuum. The sample was redissolved in 150 μL of acetonitrile:water (25:75, v/v) and filtered through a 0.22-μm filter for liquid chromatography–mass spectrometry analysis. Plant hormones were analyzed using HPLC-Q-Trap-tandem mass spectrometry system with multiple-reaction monitoring mode. Chromatographic separation was achieved on a ZORBAX Eclipse Plus C18 column (150 × 2.1 mm, 3.5 μm; Agilent). Statistical analysis was performed using one-way analysis of variance and Tukey’s post hoc test.

### XμCT Imaging

XμCT imaging of germinated date palm seedlings (stage I) was performed using a Nikon XT H 225 device (Nikon Metrology) with a Paxscan 2520DX x-ray amorphous-Si flat panel detector (Varian Imaging Systems). The XµCT device was set to operate at a voltage and current of 60 kV and 100 μA, respectively. The date palm sample was scanned at a voxel size resolution of 12 μm, with the specimen stage rotating through 360° at a rotation step increment of 0.115° over a period of ∼2 h, such that 3141 projection images in total were obtained by averaging eight frames with an exposure of 250 ms each, at every rotation step. The software CT Pro 3D 4.4.2 (Nikon Metrology) was used to perform the reconstruction of the projection images, resulting in 1524 slice images with a resolution of 1910 × 1910 pixels each. A volume rendering and analysis software (Avizo 9.2.0, FEI) was used to render images from the three-dimensional (3D) data set.

The monitoring of date palm developmental stages was performed on a Phoenix V|TOME|X M 240 high-resolution x-ray computed tomography system (GE Sensing and Inspection Technologies) at the Hounsfield Facility, University of Nottingham, UK. The scanning parameters were optimized to allow a balance between a large field of view and high resolution. The same sample was imaged at consecutive time points over 11 months. Each time, the sample was scanned with a voltage and current of 160 kV and 180 µA, respectively, at a voxel size resolution of 40 μm, with the specimen stage rotating 360° at a rotation step increment of 0.166° over a period of ∼3 h. In total, 2160 projection images were obtained by averaging three frames with an exposure of 250 ms each, at every rotation step. Because of the height of the cylinder (40 cm), five separate scans were made to cover and image the entire height of the sample. Each sub-scan was then reconstructed using DatosRec software (GE Sensing and Inspection Technologies) and then manually combined in VG Studio MAX v2.2 (Volume Graphics GmbH) and exported as a single 3D volumetric data set. To distinguish the phases of the root system from the soil material, image processing techniques were applied by segmenting the reconstructed CT data using a region-growing method in VG Studio MAX v2.2.

### Date Palm Transcriptomics

#### Date Palm RNA Extraction

Stage I cotyledonary petiole and germinated root tips (from 12-week-old seedlings) were sampled for RNA extraction. The total RNA was extracted from 75 mg (root tips) and 200 mg (embryo) of plant material. The tissue was ground at −80°C using liquid nitrogen immediately after collection. RNA isolation was performed by using TRIzol reagent (15,596,026; Ambion) according to the manufacturer’s specifications. The total RNA was treated with DNase I. RNA concentration and quality were measured by using a NanoDrop spectrophotometer and a 1.5% agarose electrophoresis gel.

#### RNA-Seq Library Generation and Sequencing

The starting material for RNA sequencing was of 10 µg of RNA per sample. Two biological replicates were used per sample. Libraries and sequencing were generated at the Genomic Advanced Unit-LANGEBIO sequencing facilities in Irapuato, México. Four independent libraries using TruSeq protocols were processed and were sequenced on a 2 × 150 platform according to the manufacturer’s instructions and recommendations.

The four libraries were sequenced in a 2 × 150 format using Illumina NextSeq platform; 47,435,916 and 49,260,416 paired end reads for the embryos and 48,058,648 and 52,789,080 paired end reads for the root were obtained (Supplemental Table 2). All reads passed our quality filters and were used in the subsequent analyses. Raw data were deposited in NCBI Sequence Read Archive under accession PRJNA497070 and can be traced on https://www.ncbi.nlm.nih.gov/bioproject/PRJNA497070.

#### Transcript Assembly and Annotation

rnaSPAdes v1.0.0 (present in SPAdes v3.9) were used to reconstruct the date palm transcriptome. Samples were combined and assembled using default parameters in rnaSPAdes. The final assembly was annotated by first identifying their main open reading frame and then comparing the peptide sequence with the NCBI Non-Redundant database (downloaded November 2015) with BLAST+ v2.2.30 and then retaining the best match with E-value < 1 e^−6^.

The resulting transcriptome contained 110,808 sequences longer than 300 bp, with an average size of 1079 bp and a maximal length of 16,237 bp of a sequence corresponding to the gene *LONGIFOLIA1*. In addition, 42,418 nonredundant mRNA open reading frames and 218,325 noncoding sequences were found.

#### Differentially Expressed Genes in Embryos

Transcript quantification was obtained using Kallisto for each library separately with a bootstrap of 1000. Effective read counts per transcript were combined in a single table and loaded in R: Bioconductor package EdgeR for detecting the embryo differentially expressed genes. EdgeR package was used to perform library normalization with computed scale factors obtained from Timmed Means for M-values normalization method. After normalization, EdgeR computed the biological coefficient of variation in samples, and it was used to estimate the relative abundance variation per gene in samples and the measurement error of the quantification. The determination of differential expression genes was computed using a negative binomial distribution to calculate a P-value for differential gene expression contrasting our conditions. The P-values obtained are fitted in an FDR adjustment to improve the results.

For downstream analyses, Arabidopsis (TAIR v11) annotation was used as reference. From the differential gene expression analysis, date palm tissue–enriched genes were used for GO term enrichment based on homolog genes in Arabidopsis. Significant GO terms were selected according to a P-value < 0.01 and an FDR < 0.05 from each tissue analyzed.

### Accession Numbers

The *P. dactylifer*a cDNA accessions used in this study are as follows: LOC103704820 (*PdSOMBRERO*); LOC103708475 (homeobox-leucine zipper protein *pdATHB-1*5); LOC103705495 (NAC domain-containing protein 78 similar to *PdCUC1*), LOC103715985 (*PdSHORT-ROOT*); LOC103711557 (homeobox protein knotted-1-like with similarity to *PdSHOOTMERISTEMLESS*), LOC103704533 (auxin-induced protein *PdIAA2*), LOC103710075 (*PdHISTONE H4-1*).

### Supplemental Data

**Supplemental Figure 1.** Germination modes in palms and rice.
**Supplemental Figure 2.** Expression analysis of genes marking the shoot meristem and organ primordia.
**Supplemental Figure 3.** Measurement of hormone contents.
**Supplemental Figure 4.** Conserved function of date palm *SHORTROOT* in Arabidopsis.
**Supplemental Table 1.** Primers used in this study.
**Supplemental Table 2.** RNA-seq reads per sample.
**Supplemental Movie.** Date palm germination and growth visualized by x-ray micro-computed tomography.

## Dive Curated Terms

The following phenotypic, genotypic, and functional terms are of significance to the work described in this paper:

SHR Gramene: AT4G37650
SHR Araport: AT4G37650
